# The effect of the Covid-19 pandemic on STEM faculty: Productivity and work-life balance

**DOI:** 10.1371/journal.pone.0280581

**Published:** 2023-01-27

**Authors:** Amanda Esquivel, Simona Marincean, Marilee Benore

**Affiliations:** 1 Department of Mechanical Engineering, University of Michigan-Dearborn, College of Engineering and Computer Science, Dearborn, MI, United States of America; 2 Department of Natural Sciences, University of Michigan-Dearborn, College of Arts, Sciences and Letters, Dearborn, MI, United States of America; Shahid Beheshti University of Medical Sciences, School of Dentistry, ISLAMIC REPUBLIC OF IRAN

## Abstract

The disruption caused by the COVID-19 pandemic impacted STEM professionals in numerous ways, affecting research, teaching, publications, patents, and work-life balance. A survey was conducted to determine the changes approximately one year into the pandemic shutdown in USA. Results indicate that the quarantine, limitations, and restrictions led to decreased work productivity and increased stress, anxiety, and family obligations. There was a significant difference between male and female faculty experience with women reporting more child-care, schoolwork assistance, and care for elderly relatives.

## Introduction

The onset of the COVID-19 pandemic in early 2020 caused numerous global changes in business and services, work capacity, medical treatment and practices, education access, economics, law and policies, and personal and family obligations and expectations. While the impact caused degrees of burden for everyone, pre-existing inequalities regarding work and family responsibilities were exacerbated [1].

The major impact of the COVID-19 pandemic to many employees was the shift to work from home across the economic sectors, although those in “essential” jobs, including hospital, food and other service sectors were required to continue in job settings, or completely lost jobs. Universities, private companies, and government institutions implemented policies regarding work amid a lack of access to campuses and institutions, with numerous employees forced to work remotely. Policies, laws, recommendations for safety and wellness along with rapid changes in knowledge and access to medications and vaccines meant the landscape shifted constantly. The restrictions and lack of access to site and services changed the work environment in STEM (science, technology, engineering, and math) where hands-on, in person activities are often critical to science and career advancement. In contrast, some service STEM workers, such as teachers, faculty, and hospital personnel were required to be on-site for work often at risk to their health.

During the COVID-19 pandemic, individuals with young and/or school age children or elderly family members had increased or new responsibilities because of school and daycare closures. Parents of school aged children may have been needed to assist with online education. Parents with jobs requiring security or privacy were forced to work at odd times or in secluded areas of the home. All these changes were taking place in an environment in which working parents already believed parenting could negatively impact work productivity, with increased fear of job loss and the concern that using parental job benefits would be viewed with derision [2,3].

Even pre-COVID-19 there were significant work and responsibility differences; among those child and elderly care on the home front, and service work are well documented. Meara et al. addressed factors such as gender, career rank, and demographics that play a role in academic service distribution [4]. Their findings confirmed earlier reports that women tend to be asked more often to participate in service to add diversity to the committee. An unfair disadvantage is generated as women are allocated time-consuming service responsibilities that include teaching committees, student recruitment, student advising, and mentorship. However, they are not able to extend their working day with “after- hours” dedicated to either the above mentioned service or to their research work impacted by the service due to family responsibilities [5–7]. Interviews conducted with 67 biology graduate students suggested that women’s laboratory research was affected by spending more time on support tasks, such as ordering reagents, and by limited access to collaborations [8]. Several analyses of preprint repositories in physical and life sciences, astrophysics and economics led to similar findings: during the pandemic the women-authored preprints decreased at a higher rate than the ones deposited by male scientists. The differences were more pronounced for first authors raising concerns for long term effects of early career researchers [9]. Other studies have examined the impact of the pandemic on scientific output and found there have been various gender differences in the effect on work productivity, stress, and increased domestic workload [10–15].

The number of women and minority professionals in STEM fields varies. Some fields, such as biology and math education, show near equity in women receiving undergraduate or advanced STEM degrees, while in others, like engineering, there are disproportionately fewer seeking these degrees or advancing through degree or promotional ranks in universities or in positions of leadership in industry, with 21%, 25%, and 24% receiving BS, MS, and PhD degrees, respectively [4]. Although more programs have been instituted across campuses, the proportion of minority students that earn STEM BS degrees is at 22% decreasing to 13% for MS, and 8% for PhD. Moreover, STEM employment among minorities is around 20% with even lower values for the academic sector, at 10% [4]. The STEM workforce is negatively impacted when capable individuals are shut out or lost from opportunity. Statistics gleaned from international sources paint a similar picture of the number of women choosing, then leaving STEM careers. Additional responsibilities and roadblocks caused by the pandemic could exacerbate this problem.

Our hypothesis was that the COVID-19 pandemic negatively affected productivity and work-life balance for STEM faculty and that the negative effects were more pronounced for female faculty. The aim of the survey was to identify the impact of the COVID-19 pandemic on the productivity (papers, grants) and ability to perform critical aspects of the work such as data collection from experiment design and performance, collaborative work, students recruitment and supervision, of those in the STEM fields in the United States of America (USA). The focus on USA STEM faculty was due to the numerous differences that were apparent in the pandemic handling, medical treatment, and workplace policy mandates in the USA versus other nations. Furthermore, while this will no doubt impact young researchers, especially parents, globally, the situation is intensified in nations such as the USA where parents rely on day care, with few options for paid family leave, and inequitable health care coverage. By surveying individuals, evaluating, and benchmarking the issues and concerns, the extent of the problems can be identified and measured, leading to strategies for solutions.

## Materials and methods

### Survey

Prior to commencement of the study, the survey questions were written and tested for anonymous and non-anonymous feedback by a small group, to ensure clarity and completeness, and responses used to adjust survey questions. The questions were set up to either generate specific responses, which were sub-linked for further detail, or open-ended. Most questions required a response. The survey was intended for STEM professionals in the United States.

IRB approval was secured from the University of Michigan (HUM00189064). The survey was written and posted via Qualtrics. 3098 emails were distributed via Qualtrics, the majority with anonymous survey links. Different verbiage was used for emails: one email designed for Chairs with a request to redistribute, one to individuals in the contact list. These emails, meant to be forwarded, also ensured the recipients received an unlinked site to visit, and thus were anonymous. Since a pre-pandemic survey was not conducted due to the nature of the pandemic, questions attempted to elicit responses that were comparative.

The survey questions were organized in the following categories:

- demographics: age, gender identity, race, ethnicity, and country of birth
- education: degree type and date earned, STEM area
- employment: field, employer information, title, job duties
- measures of productivity: research, grants, patents and site/workplace/lab access
- work/life balance: responsibilities and duties

Questions regarding productivity and work/life balance queried pre and post COVID-19 experiences. Many questions solicited optional comments and the relevant ones are presented as a table.

### Participants

A contact list was created *de novo*. Two main sources were used: NSF grant sites and The Rutgers Center for Minority Serving Institutions, to generate a list of academic institutions. The surveys were sent to individuals at 1600 distinct institutions. The list was created by using a combination of colleges and universities taken from several institutional databases, to ensure we solicited from research universities, predominantly undergraduate institutions, and campuses which are more likely to have minority or faculty of color. From granting agencies we selected several universities with well funded STEM grants and combed the web sites selected names and email addresses. For predominately undergraduate and primarily minority institutions we used lists created by similarly researching science faculty. The same was true for ensuring primarily minority institutions. There were most likely fewer representatives from industry and hospitals, except our personal network contacts and their network, as these contacts are not typically available online to the public.

The response rate was low and decreased over time in the summer months.

No minors were contacted, and question included specific degree information to document that college or advanced degrees were required. The individual contacts were collected from the STEM departments’ websites at those institutions. A similar group of names was obtained from select industry websites, but access to STEM individuals was less available.

Data were collected from participants across the United States, May through Mid- July 2021. The response rate varied from approximately 10% in May to 3% in July.

No compensation was provided to participants. A follow-up second email was only sent to the last cohort of ~300 names.

### Survey assessment and statistical analysis

Descriptive statistics were compiled for demographic data including information about the sex, age, race/ethnicity, level of education and type of job position. Data comparing pre- and post- COVID-19 reported productivity and time spent caring for family were analyzed by a one-tailed paired Student’s t-test. If participants left responses blank, they were omitted from analysis. For example, if they never submitted patents before or post the COVID-19 pandemic, the data was not included in the tallies. Reported decrease in productivity and increase in time spent caring for family members were compared between men and women using a two-tailed unpaired t-test. A Chi-Square Test of Independence was conducted to test for an association between variables that reportedly affected work productivity and gender (male or female). All differences were considered significant if p < 0.05.

## Results

There were 158 total responses to the survey. As shown in Table 1, the majority of the survey participants were female (61%), white or Caucasian, not Hispanic (76%), with a doctoral degree (79%), and employed at a university (78%). For most of the participants the age was in the 35–64 years range, with a fair split among the decades, Table 1.

**Table 1:** Respondent demographic information.

Gender	
Female	61%
Male	37%
Non-Binary	1%
Prefer not to say	1%
Age	
Under 21	0%
21–34 Years	13%
35–44 Years	36%
45–54 Years	22%
55–64 Years	22%
65–75 Years	6%
Over 75 Years	1%
Race/Ethnicity	
American Indian or Alaska Native	0%
Asian	9%
Black or African American	1%
Native Hawaiian or Pacific Islander	1%
White or Caucasian, not Hispanic	76%
White or Caucasian, Hispanic	9%
Other	5%
Educational Background	
Doctoral Degree	79%
Professional Degree (JD, MD, etc.)	4%
Master’s Degree	9%
Bachelor’s Degree	6%
High School Graduate	0%
Associate Degree	0%
Some college but no degree	1%
Employment Institution Type	
University	78%
Government Agency	1%
Private or Public Company	10%
Self-Employed	2%
Other	1%
Hospital	7%

When examining the data, there was a significant decrease in reported published manuscripts, grants and conferences attended when comparing the number of each pre-COVID-19 to post-COVID-19 (p<0.032). The average number of published manuscripts per year was reduced from 2.46 to 1.80; the average number of grants submitted decreased from 1.97 to 1.14 and the average number of conferences attended decreased from 2.52 to 0.95. There was no significant difference in patent applications; however, very few respondents indicated that they typically submit patents (Table 2). We did not find any gender effect on dissemination and grant seeking activities.

**Table 2:** Participant reported average number of manuscripts, grant applications, conferences attended and patents pre- and post- COVID-19.

	Published Manuscripts		Grant Applications		Conferences Attended		Number of Patents	
	Pre-	Post-	Pre-	Post-	Pre-	Post-	Pre-	Post-
Average	2.46	1.80	1.98	1.15	2.52	0.95	2.80	2.00
Standard Deviation	3.20	3.52	2.57	1.62	1.85	1.31	6.09	2.91
P-value	0.032		0.002		<0.001		0.235	

Most participants indicated that limitations on time spent in the office and the limitation on the number of people allowed in a space affected their productivity (Table 3). In addition, for female STEM professionals, a majority indicated that stress and anxiety due to the pandemic also affected productivity and there was a significant association between gender and stress and/or anxiety related to the pandemic (χ2 = 7.408, p = 0.006) (Table 3). There were no other significant associations.

**Table 3:** Factors that affected productivity.

	Office time limitation	Space occupancy limitation	Human subjects research limitation	Laboratory time limitation	Scheduled space sign up	Workplace imposed travel restrictions	Quarantine requirement
Male	61.0%	54.2%	18.6%	37.3%	16.9%	49.2%	27.1%
Female	51.0%	51.0%	16.7%	33.3%	13.5%	42.7%	30.2%
c2	1.181	0.068	0.099	0.135	0.336	0.43	0.299
p value	0.277	0.794	0.753	0.713	0.562	0.512	0.585
	Increased teaching	Additional duties	Equipment or supplies delays	Technology challenges or limitations	Sickness due to contracting COVID-19 myself	Taking care of a friend or relative with COVID-19	Stress and/or anxiety related to the pandemic
Male	16.9%	33.9%	28.8%	33.9%	5.1%	6.8%	39.0%
Female	27.1%	25.0%	28.1%	42.7%	6.3%	5.2%	60.4%
c2	2.105	1.095	0.002	1.478	0.091	0.165	7.408
p value	0.147	0.295	0.962	0.224	0.763	0.685	0.006

For participants who indicated that they spend time caring for children, assisting children with schoolwork, caring for elderly family members, and conducting housework, we found that this amount of time significantly increased post-COVID-19 compared with pre-COVID-19 (p<0.0116) in all four of these categories (Table 4).

**Table 4:** Reported number of hours per week spent caring for children, assisting with schoolwork, caring for elderly family members and housework pre- and post- COVID-19 for all subjects.

	Care for Children (hours/week)		Assist Children with Schoolwork (hours/week)		Care for Elderly Family (hours/week)		Housework (hours/week)	
	Pre-	Post-	Pre-	Post-	Pre-	Post-	Pre-	Post-
Average	20.70	33.51	3.57	10.78	2.93	6.83	7.44	10.11
Standard Deviation	16.56	24.16	2.62	9.41	2.56	9.52	4.50	5.58
P-value	<0.001		<0.001		0.016		<0.001	

When comparing the increase in time spent on helping children with schoolwork and housework, female respondents reported a much greater change than males (p<0.046). Female respondents had a larger increase in time spent on childcare (p = 0.134) and caring for elderly relatives (p = 0.085) but this was not significant (Table 5).

**Table 5:** Reported change in the number of hours per week spent caring for children, assisting with schoolwork, caring for elderly family members and housework pre- and post- COVID-19 for males and females.

	Change in Hours Per Week Post-COVID-19 versus Pre-COVID 19							
	Child Care		Help with Schoolwork		Care for Elderly Family		Housework (hours/week)	
	Male	Female	Male	Female	Male	Female	Male	Female
Average	8.21	14.12	3.17	9.78	0.62	6.56	1.76	3.29
Standard Deviation	13.69	16.12	5.23	9.05	2.29	11.77	3.39	4.67
P-value	0.134		0.002		0.085		0.046	

## Discussion

The data clearly indicate that STEM researchers were negatively affected by the pandemic and resulting shut down, particularly in specific areas critical to their work and future success. Comparisons of pre- and post- COVID-19 analysis indicate that fewer papers were published, fewer conferences attended, and fewer grant applications were submitted which validates the general public concern and our hypothesis that COVID-19 would have a negative effect on reported “research productivity” (as measured by published manuscripts, grant applications, conference participation and patents received). Results from the survey were consistent with several published studies regarding disruption due to COVID [16–18]. There was insufficient data to compare to those who did not report gender.

Due to the nature of this anonymous survey, we cannot determine the bias in the response rate. We do note in the article that survey invitation emails were sent to individuals as well as from our bespoke list, as described. We do note that two thirds of the responses were from women. It is unclear why this occurred. However, it may be due to the nature of the impact of the pandemic, and a wish by women to comment on their situations and work.

Collected data indicated that all faculty, regardless of gender, experienced limitations on time spent in the office or laboratory due to workplace policies regarding pre-registration for in-person work, number of people allowed in a working space, and ability to conduct human subject research. In addition, work travel restrictions, and quarantine requirements were put in place across the nation. All these new regulations led to loss of laboratory or office work time although the overall workday increased [19] with a long-term impact on professional careers. While fewer respondents reported as males than females, we also compared the decrease in productivity between men and women and found that there was no significant difference in the reported decrease in productivity between the genders, potentially due to our small sample size. While other components of STEM professional jobs are important, they are less likely to be considered in the success of an individual in the promotion and tenure process. In most STEM fields, research productivity is still the measuring tool of success [20].

The most significant and concerning gender linked variation pre and post COVID-19 was the amount of time spent on duties associated with family and personal care. In all these categories, caring for children, assisting children with schoolwork, and caring for elderly family and housework significantly increased post-COVID-19 compared with pre-COVID-19. The estimated increased hours per week were over 20, on top of work and regular pre-COVID-19 levels, with a heavier burden on women. Assisting children with schoolwork was an area where female respondents experienced a greater change than males. Workplace policies that assist parents of school aged children may help alleviate some of this burden.

Stress was significantly heighted for women, and while nearly half of participants indicated increased stress and anxiety post COVID, women were 50% more likely than men to experience it. While some respondents indicated they appreciated and found online work to be successful, most related concerns regarding the long-term security of this kind of job.

The results from our work are consistent with other studies. While this study was sent to a broad group of STEM professionals in academics, and some to industry and hospitals, the majority of the responses were from academic faculty. As reported in the data, few responses were from industry or hospitals. Since over 1600 distinct institutions were contacted, the majority academic, we believe that response rate was consistent with the contact list. A study on academic productivity among STEM faculty in the first two months of the COVID-19 pandemic found that women faculty and those with young children at home submitted fewer papers, while productivity remained similar or increased for the other faculty categories. One reason might have been the reduced number of working hours, around 15 hours/week less during COVID-19, due to the demands of home childcare. A limitation of the study was the under representation of some races [14].

A survey of 362 US university biologists, biochemists, civil and engineering faculty on the COVID-19 impact on their scientific productivity analyzed the differences based on gender, rank and pandemic “hot spots”. Among their reported major negative effects, regardless of the gender of faculty, were lab work disruption, lack of access to research, lack of students’ participation, and ability to establish collaborations. While both women and men report the same ranking order of factors that affect their ability to focus on research, nearly twice as many women than men expressed that the lockdown negatively impacted their research time. Our data correlated with those findings. A positive impact of the stay-at-home situation was the opportunity to explore new research. Analysis of the responses’ distribution suggested that female and assistant professor faculty were overrepresented while associate and full professors were underrepresented [12]. In contrast, our respondents were more likely to be more advanced in their careers, but the results were similar.

We observed a slight decline between men and women in the scholarly output, consistent with published reports [13]. In a survey of STEM professionals in Spain on COVID-19 impact based on gender, Rodriguez-Rivero et al. compared the results to pre COVID-19 data [15]. Before the pandemic, women perceived that 35% of their free time was spent on household activities while men reported that both themselves and their partners dedicated less than 20%. Upon lockdown, the respective times increased to 50% (women) and 35% (men), correlated to the number of children for the former unless the women had the highest income in the household [15]. Despite the negative effects of the lockdown (isolation, relative death, parents becoming teachers with no outside support for childcare) the respondents expressed a desire to work from home following the pandemic. Our data, collected later, indicated that actual scholarly output measured as grants, papers, and patents was altered.

We have reported the comments as they provided insight into the individual impact of COVID-19 on their lives, stress, and concerns about their futures. (S1 Table) The comments were sorted into the categories of concern as: Career Expectations, Emotional, General Concerns, Workload, Frustrations, Future Concerns, and Positive Outcomes. The respondents identified the disruption as a delay, expressing worries over lack of productivity compared to pre-COVID-19 expectations, with long term impact on collaborations and funding. For some, the apparent productivity was maintained due to the lag between data collection and publication, but future data generation was a source of concern. The psychological impact described in the comments was caused mainly by faculty supporting role for students and increased teaching load. Some researchers expressed frustration against perceived lack of support from their institution and the absence of childcare.

Respondent comments were consistent with the views expressed in previous studies. Interviews conducted with Canadian faculty regarding professional and personal impacts experienced during COVID-19 showed common themes with those in our study such as: faculty feeling overextended with learning new virtual technology, changes in teaching style, increased need to support students, and unmet obligations as research scholars. These effects were more prevalent in minority groups due to reduced presence of support groups [11].

Our study validated our hypothesis that while career and workload were impacted by COVID-19, women were more likely to be burdened by additional housework, child, and elderly care as result of the COVID-19 pandemic, tasks that were reported as frustration generators regardless of identified gender or family role. Future initiatives to improve the workplace should consider that gender disparities are enhanced by emergency situations such as the COVID-19 pandemic that have a long-term impact.

These results and other studies clearly demonstrate that STEM professionals were adversely affected, and their careers impacted by the pandemic. This is especially damaging given that more women are lost as defined by the “leaky pipeline.” Numerous calls to action, that go beyond awareness, indicate the need to create binding policies to combat the inequity, and support these STEM professionals [21–23]. This will not happen without support from professional societies, university and government recommendations, and broad awareness of the severity of the problem and danger of the outcome if ignored [24].

## Supporting information

S1 Table Comments from respondents. (DOCX)
