# Supplementary material for: The effect of the Covid-19 pandemic on STEM faculty: Productivity and work-life balance
Source: PLoS One. 2023 Jan 27;18(1):e0280581. doi: 10.1371/journal.pone.0280581 (PMC9882624; doi:10.1371/journal.pone.0280581)
Supplement: S1 Table — (DOCX) [file pone.0280581.s001.docx]

**S1 Table. Comments from Respondents**

| **Impact** | **Comment** |
| --- | --- |
| Career Expectations | I’m very concerned that others won’t properly calibrate their expectations for career related accomplishments to reflect the pandemic |
|  | My lack of productivity during the pandemic caused many of my ongoing collaborations to end prematurely and for many of my collaborators to move on to projects that don’t involve me. I fear that this will have a lasting negative effect on my research career |
|  | I’m trapped in an adjunct instructor job. In American academia that is not a career, it’s a dead-end job. |
|  | Want to retire. Moving retirement date forward. It is too hard to teach well during covid. Have reconsidered priorities. |
|  | It’s caused me to re-evaluate my role in direct patient care. I am actively engaged in pursuing consulting rather than patient care or research. |
|  | My research students were at home studying remotely so in-lab work suffered |
|  | Some productivity improvements (work-from-home and video meetings). |
|  | It was the catalyst for me changing my career and starting a new business, becoming self employed |
|  | My lack of productivity in publishing papers may negatively affect funding for my department as well as negatively affect review of my grants |
|  | loss of social interaction on the job which indirectly caused a loss in productivity |
|  | Pushed my life/work timeline back. As a female postdoc in “prime” child-bearing years, I was hoping to have settled in a more long-term job in the next year prior to trying for kids. Now it feels like my plans have been pushed back 1-2 years which is stressful from a family planning perspective. |
|  | I’m considering changing fields and pursuing a nursing/public health degree |
| Emotional | The decreased contact with live people has affected mental and emotional well-being. |
|  | It has been very isolating to work from home without normal workplace interactions with colleagues & students |
|  | Emotional labor of supporting and mentoring students, all of whom really struggled both academically and personally because of COVID impacts, took a big toll on my emotional health and my work time this academic year |
|  | Current framework is not sustainable in terms of being a parent, Professor, and researcher. |
|  | Languishing has made it much more difficult to focus on work and be innovative. It’s hard to just maintain status quo. |
|  | Work life balance was destroyed by the work from home shift. |
|  | Travel ban makes it impossible to visit other academic places for future collaboration. Covid pandemic makes it much more difficult to take care of chronic health problems, like heart attacks. I have to wait for months to get a specialist and another month for a test. It definitely delays the treatment at the right time and cause worse symptoms which stop my research and teaching. Visiting families becomes impossible due to travel ban and risks of covid. This stress makes it harder to keep work efficient. |
|  | I’m a mother, and my young child had to be home for 1.5 years. I had to monitor her online schooling while continuing my faculty position. We were at home because of my high-risk status for COVID complications. |
| General Concerns | It has affected students more than anybody else |
|  | I am lucky to be post-tenure (full professor) and can relax more about lack of productivity. I feel for pre-tenure faculty who are clearly stressed. I also feel as if I was able to increase my publications because former students found themselves with more time and returned to publications. |
|  | I got through this year with relatively little disruption to my research because I had students working with me who were already trained. Next year, I will welcome students into my research group who are not trained and some who spent the last year remote, so their lab skills will be severely limited compared to a normal year. That will increase the amount of time I need to spend training them and it’s likely to decrease their productivity. |
|  | The negative effects are not so apparent now because papers we published in 2020 and early 2021 are based on data that was mostly collected pre-COVID. Now going forward, I have nothing more to publish and limited preliminary data for grant applications, and this will show in reduced productivity next year and in subsequent years. I anticipate struggling to get funding as a result. It was great that NIH allowed us to continue paying students and technicians during the shutdown, but that means that time and money is simply lost - to get the science done, I need more time and money. No cost extension is not good enough, we need funds to continue to pay lab staff. |
| Workload | Hugely increased my workload. Switching to online teaching was intense. Now that I have things in place, it’s a bit easier. I will teach Fall online again, but then move back to F2F in Spring. I expect the transition to be a challenge again, trying to get back into the swing of things in person! |
|  | I had to care for more students as many were ignored by their advisors who became unresponsive. |
|  | Feeling more interested in hybrid work environment- wfh 2-3 days sounds great |
|  | I did not have children at home to care for. I think the pandemic improved my career because I spent less time commuting and was able to access more professional development opportunities due to the ease of online attendance at trainings/conferences. |
|  | A lot more work to accommodate both remote and in person students. As Department Chair MUCH more work making sure department runs well. Administrative duties greatly increased |
|  | Biggest effect of COVID was in how my classes were delivered (hybrid), and the amount of time put into implementing those changes. |
|  | There is no work/life division anymore. For faculty, there never was, but my wife now routinely works weekends and she is not faculty. Guess who takes care of our son when she’s working? |
|  | Delayed obtaining childcare for my infant son by 6 months due to pandemic, so instead of doing sabbatical work I was the full-time caregiver for my son |
|  | Has forced me to make 180 degrees switch in my research, to something that could be done remotely. |
|  | Huge amount of time spent transforming curricula to 100% online teaching format (creating new assignments, recording YouTube video lectures). Doing all assignment grading myself because university funding for grad student TA employment was massively cut |
|  | My children were out of school and my husband went back to work. I needed to care for them AND teach them while trying to keep up with my regular work. |
|  | Inefficient administration of my department due to a lack of in-person work. |
|  | Lack of in-person interaction. Problems creating group atmosphere while online. Increased burden of home maintenance work. University relying on faculty to volunteer for menial jobs (distributing PPE) as well as covid-related research and service |
| Future concerns | Due to covid it has been hard to recruit student researchers and there will be a lack of trained work force in my lab for years to come, I am sure this is a significant problem for others but was not addressed here |
|  | It has been very difficult to recruit graduate students to join my Research program due to US High Commissions being shut down during the pandemic. |
|  | I think the effects of the pandemic, the slow-down in research productivity, will linger for the next couple of years, and I hope my institution considers this even as restrictions are eased. |
|  | Kids are not in school without access to child care. Full shut down of the research laboratory for 7 months. |
| Frustration | Taught all my classes via Zoom remotely from my garage with little direct student or staff contact. |
|  | The lack of focused time, together with a feeling of disconnection from colleagues, has made things like writing papers and grants much more difficult. |
|  | Disappointment with the public health authorities at all global, federal, state levels |
|  | The COVID pandemic was only one of the pandemics we’ve contended with recently. Asking about the effects of the pandemic without also considering political corruption, racial injustice and rising hate crimes (and not limited to race), economic crisis, etc. can only capture a piece of the overall picture. The pandemic was a massive disruption within a thoroughly broken/faulty system of injustice |
|  | Disparity in lab reopening and social distancing across the country definitely puts some universities and even labs at the same university at a significant disadvantage. |
|  | Fall 2020 we had in-person classes, but used a de-densification strategy that was miserable and time-consuming. That semester was brutal and there was no time for research on top of the class preps. |
|  | My husband’s father was our main source of child care. With the start of the pandemic, we couldn’t have him care for the kids due to risk of him contracting COVID. Though my husband is only part-time, it made child care incredibly stressful and challenging! |
|  | With schools closed for in-person school in our district, childcare was a huge increased burden, greatly reducing the amount of time I had to spend on research and teaching tasks. |
|  | Having to deal with Elementary School age children having to home-school while trying to work remotely. |
|  | Lack of childcare and in-person schooling for elementary-aged child for an entire year |
|  | lack of childcare!!! |
|  | Lack of in-person interaction. Problems creating group atmosphere while online. Increased burden of home maintenance work. University relying on faculty to volunteer for menial jobs (distributing PPE) as well as covid-related research and service |
| Positive outcomes | I love teaching from home and hope to continue. |
|  | I received an accommodation to work from home throughout the pandemic. The biggest change will be the loss of time having to now drive to campus to teach and attend meetings. |
|  | While I have been working from home all this time, my company introduced informal ways of communicating where I connect with colleagues from all over the world during a “coffee corner”. I really enjoy these moments, sharing experiences with people from other countries, other continents, but where we are all connected as humans |
|  | the advent of zoom has made admin much easier |
|  | Student internship (though drastically cut during 2020) have increase remote options and therefore I have been able to apply to more opportunities that do not require relocation. |
|  | I was on sabbatical for the year of COVID. While I wasn’t able to work in the lab, I was able to write up papers and finish some projects. |
